# Aging Well, Aging Together: Experiences of Loneliness and Community Connection for Older Lesbians

**DOI:** 10.1093/geroni/igaf122.002

**Published:** 2025-12-31

**Authors:** Christine Happel, Joanne Patterson, Julia Applegate

**Affiliations:** The Ohio State University, Columbus, Ohio, United States; The Ohio State University, Columbus, Ohio, United States; The Ohio State University, Columbus, Ohio, United States

## Abstract

Older lesbians in the U.S. face challenges to aging well, including health disparities and lack of culturally competent medical care. However, older lesbians are recognized as an “at-risk, yet resilient” population. Enhancing resiliency requires understanding how protective factors like community and social engagement promote aging well in the lesbian community. This cross-sectional, qualitative study was designed to: (1) understand the experiences of loneliness among older lesbians in Central, Ohio (2) examine the impact of the disappearance of lesbian social spaces; and (3) identify strategies to support aging well for lesbians. Interviews were conducted with individuals age 55 + (N = 34); who identified as lesbian/woman loving woman/gay/queer; and spoke English. Forty percent of participants were ages 70+, 25% were people of color, 53% lived alone, 85% attained at least an undergraduate degree, and 94% still drove. Interviews were analyzed using the team-based flexible coding method. Few interviewees identified as lonely, but most expressed dissatisfaction with their social relationships. Lesbian bars impacted social capital and identity exploration in interviewee’s youth but not in later life. Interviewees collectively expressed pride in their lesbian identity, shared stories of past advocacy, and felt no pressure to conform. Small, mostly non-lesbian community groups, and friendships were identified by interviewees as stress-reducing supports. Few interviewees had a formal network for instrumental support beyond their partner or, in some cases, children. Lesbian-specific shared living communities; culturally congruent assisted and nursing care; accessible transportation; and a centralized community center were identified by participants as solutions for supporting aging well.

